# Semaphorin 7A restricts serotonergic innervation and ensures recovery after spinal cord injury

**DOI:** 10.1007/s00018-020-03682-w

**Published:** 2020-10-30

**Authors:** Kristina Loy, Julie Fourneau, Ning Meng, Carmen Denecke, Giuseppe Locatelli, Florence M. Bareyre

**Affiliations:** 1 Institute of Clinical Neuroimmunology, University Hospital, LMU Munich, 81377 Munich, Germany; 2 Faculty of Medicine, Biomedical Center Munich (BMC), LMU Munich, 82152 Planegg-Martinsried, Germany; 3 Graduate School of Systemic Neurosciences, LMU Munich, 82152 Planegg-Martinsried, Germany; 4 Munich Cluster of Systems Neurology (SyNergy), 81377 Munich, Germany

**Keywords:** Locomotion, Patterning, Recovery, Semaphorin7A, Serotonin, Spinal cord injury

## Abstract

Descending serotonergic (5-HT) projections originating from the raphe nuclei form an important input to the spinal cord that control basic locomotion. The molecular signals that control this projection pattern are currently unknown. Here, we identify Semaphorin7A (Sema7A) as a critical cue that restricts serotonergic innervation in the spinal cord. Sema7A deficient mice show a marked increase in serotonergic fiber density in all layers of the spinal cord while the density of neurons expressing the corresponding 5-HTR2α receptor remains unchanged. These alterations appear to be successfully compensated as no obvious changes in rhythmic locomotion and skilled stepping are observed in adult mice. When the system is challenged with a spinal lesion, serotonergic innervation patterns in both Sema7A-deficient and -competent mice evolve over time with excessive innervation becoming most pronounced in the dorsal horn of Sema7A-deficient mice. These altered serotonergic innervation patterns correlate with diminished functional recovery that predominantly affects rhythmic locomotion. Our findings identify Sema7A as a critical regulator of serotonergic circuit formation in the injured spinal cord.

**Electronic supplementary material:**

The online version of this article (10.1007/s00018-020-03682-w) contains supplementary material, which is available to authorized users.

## Introduction

Serotonin (5-HT) is a monoamine neurotransmitter synthesized by a subclass of neurons termed serotonergic neurons that are present in the brainstem. In higher vertebrates, locomotion is modulated by descending monoaminergic input onto spinal central circuits [1]. Specifically, there are three different aminergic systems that project to the spinal cord: serotonergic (5-HT), noradrenergic (NA) and dopaminergic (DA). The 5-HT tract system is not only the most widespread one in the mammalian CNS but also the oldest phylogenetically and ontogenetically [2]. The vast majority of 5-HT profiles in the spinal cord originate within the caudal group of the raphe nuclei in particular from the raphe obscurus, raphe pallidus, raphe magnus and ventral lateral medulla with most of the neurons of origin in the raphe obscurus [2]. In adult, 5-HT axon terminals can be observed at all segments of the spinal cord [3, 4] and are localized throughout the spinal grey matter in particular in specific regions of the dorsal, ventral and intermediate layers of the spinal cord [5, 6]. Several groups have demonstrated that 5-HT regulates locomotion, postural muscle tone, rhythm and coordination of movements via spinal interneurons and the direct contacts to 5-HT receptors located on motoneurons [5, 7, 8]. This 5-HT has been shown to control spinal reflexes, motor function and hindlimb coordination by increasing spinal motoneuron excitability directly but also indirectly through its effects on spinal interneurons [9–11]. The precise patterning and targeting of serotonergic inputs onto lumbar motor networks is therefore essential for efficient locomotion but also for recovery of locomotion following injury. How this precise patterning of serotonergic fibers is established in development and adapted after injury is therefore key to the understanding of locomotion and its recovery.

The correct wiring of CNS circuits during development is controlled by a wide range of guidance molecules (for review [12]). While circuits are established, these molecules tighten and precisely control the organization, growth and navigation of axons along what will become their final path. Semaphorins are a family of such molecules that have been shown to play an important role in guiding growing axons and controlling plasticity of synaptic connections not only during development but also in response to pathological insults [13]. Semaphorin 7A (John Milton Hagen Blood Group antigen, Sema7A), a membrane-anchored protein, affects axonal growth and axonal pathfinding/targeting via its interaction with its receptors plexinC1 (Plxnc1) and α1β1 integrin (very late antigen-1; VLA-1) respectively [14–16]. In the nervous system, Sema7A controls axon outgrowth in the lateral olfactory tract (LOT) [15] and loss of Sema7A was shown to result in aberrant patterning of the barrel cortex [17] and impaired climbing fiber elimination in the cerebellum [18]. Sema7A signaling was also recently reported to induce activity-dependent olfactory synapse formation during the neonatal period [19]. In addition to these well-established roles in promoting axon outgrowth and in regulating synapse formation and elimination, Sema7A was recently also shown to control the precise targeting of monoaminergic axons, in particular, the segregation of nigrostriatal and mesolimbic dopaminergic pathways [20]. Because Sema7A is not only expressed in the developing spinal cord [21], but is also present in adulthood all through the different laminae of the spinal cord [22], we hypothesized that Sema7A signaling could be an important molecular cue that instructs the patterning of monoaminergic inputs to spinal locomotor networks.

Here we used Sema7A deficient mice [15] to investigate if Sema7A is required for the correct targeting of descending serotonergic pathways in the lumbar spinal cord. We found that Sema7A deletion resulted in a more than twofold increase of the serotonergic innervation that equally affected the ventral, intermediate, and dorsal layers of the lumbar spinal cord of adult mice. Of note, locomotion and gait analysis of Sema7A deficient animals did not show any abnormalities indicating that the spinal locomotor circuits can adapt to the excessive but balanced serotonergic input. However, when the spinal circuits are challenged by a traumatic lesion, the reorganization of serotonergic innervation leads to an altered patterning of serotonergic input in Sema7A deficient mice that is accompanied by impaired recovery of locomotor function. Our study identifies Sema7A as an important restrictive cue for serotonergic innervation of the injured spinal cord.

## Materials and methods

### Animals

Adult female and male Sema7A knock-out mice (Sema7A^−/−^, The Jackson Laboratory Sema7A^tm1Alk^/J #005128 [15],) from 6 to 8 weeks of age at onset of the experiments were used in this study. All animals were housed with a 12 h night day cycle and received food and water ad libitum. All animal procedures were performed according to institutional guidelines and were approved by the local authority (Regierung von Oberbayern #ROB-55.2-2532.Vet_02-15-135).

### Surgical procedures

For all surgical procedures mice were anesthetized using an intraperitoneal injection of a midazolam/medetomidine/fentanyl mix (MMF; midazolam 5.0 mg/kg, medetomidine 0.5 mg/kg, fentanyl 0.05 mg/kg) and kept on a 38 °C heating pad until fully asleep. After surgery, all mice were returned to the heating pad and kept there until fully awake. For pain management meloxicam (Metacam^®^, Boehringer Ingelheim) was administered before and every 12 h after surgery.

#### Dorsal thoracic hemisection

Dorsal thoracic hemisections were performed as previously described [23–25]. In brief, the skin above the 8th thoracic vertebrae was incised and the bone was carefully exposed. A laminectomy was performed, followed by a dorsal hemisection of the spinal cord with small iridectomy scissors leaving the ventral grey and white matter intact.

#### Stereotactic labelling of the hindlimb motor cortex

In brief, the skin above the skull was incised and a hole was drilled based on the stereotactic coordinates. One microlitre of 10% biotinylated dextran amine (BDA, in 0.1% phosphate buffer (PB), 10,000 MW, Thermo Fisher Scientific D1956) was pressure injected in both hindlimb motor cortices 14 days prior to sacrifice with a finely pulled glass micropipette. Coordinates were − 1.3 mm rostro-caudal, 1.1 mm lateral from bregma and 0.6 mm depth. The micropipette remained in place for 3 min following the injection.

#### Labelling of long propriospinal neurons

Long propriospinal neurons (LPSN) were labelled using 0.5 µl of 2.5% dextran tracer conjugated with TexasRed^®^ (3000 MW, Thermo Fisher Scientific D-3328) in both sides of the cord between the last thoracic and the first lumbar vertebrae. First, the skin was opened and the gap between the two vertebrae was exposed. The dura was carefully opened and the TexasRed^®^ pressure injected with a fine glass micropipette (lateral from the central vein: ± 0.6 mm, 0.8 mm depth) which was kept in place 3 min after the injection. Animals were sacrificed 9 days after labelling.

#### Backtracing of the thoracic and hindlimb motoneurons in the cortex

Thoracic and hindlimb motor cortex neurons were backtraced from the 8th segment of the spinal cord using 2.5% TexasRed^®^. After a dorsal hemisection, as described above, a finely pulled glass micropipette was inserted rostral to the lesion and ± 0.2 mm lateral from the central vein and at 0.2–0.3 mm depth, thereby labelling the cut dorsal CST. The micropipette remained in place for 3 min after the end of injection. Animals were sacrificed 10 days after injection.

### Behavioural evaluation of hindlimb stepping and coordination

All animals were habituated three times to all equipment prior to the study. Animal with poor performance at baseline or incomplete lesions of the dorsal and dorsolateral CST defined anatomically after perfusion were excluded from the study. Ladder rung and rotarod tests were performed prior to injury (baseline) and up to 56 days after SCI. Catwalk^®^ and hindlimb clasping tests were performed prior to injury and up to 21 days following SCI.

#### Ladder rung test

For assessment of regular walking and fine paw placement the ladder rung test (also called gridwalk test) was used [26]. In this test, the animals had to cross a 1 m horizontal grid ladder and footfalls were counted by an investigator blinded to genotype and time point based on video recordings frame-by-frame of three consecutive crossings. We evaluated rhythmic paw placements in the regular walk task with evenly distributed spacing between the rungs and the animal’s ability for fine coordinated paw placements using irregular spacing of the rungs (irregular walk task). Only consecutive steps of the hindlimbs were analyzed. Therefore, the last step before or after any interruptions were not scored. Placements were considered as a “miss” when mice either totally missed a rung or if they slipped from a rung (deep or slight slip). Placements were considered as correct when the mice correctly placed all the foot or only a portion of the foot on the rungs. Then the number of slips over a standard distance was calculated quantitatively.

#### Rotarod test

To evaluate motor ability, we used the rotarod test (Ugo Basile, Italy). Mice were placed on the apparatus that was either kept a constant rotating speed of 20 rounds/min (constant RotaRod) or accelerated from 2 to 40 rounds/min (accelerated Rotarod). Maximum score was 120 s, the device automatically recorded the time and velocity at which the mice fell from the rod.

#### Hindlimb clasping test

To evaluate hindlimb clasping which is an early indication of the possible development of spasticity following injury, the mouse was held by its tail and the posture of the hindlimbs were examined. The mice were rated 0, 1 or 2, with 0 representing a perfect splay of the hindlimbs up to a defined horizontal line. They were rated 1 when their hindlimbs splayed below the horizontal line and 2 if they were unable to spread their hindlimbs outward [27].

#### Catwalk^®^ gait analysis

We performed gait analysis with the Catwalk XT™ system ([28] Noldus). For data collection, all animals were familiarized with the system three times prior to pre-injury acquisition. For each animal, three valid runs for each time point (pre-injury, 2 dpi and 21 dpi) were recorded. A run was considered valid, if it fulfilled the requirements pre-set in the system, a minimum run duration of 0.5 s, a maximum run duration of 4 s, and a maximum speed variation of 60% to ensure that the animal walks on the runway without interruptions.

The Catwalk^®^ generated more than 100 parameters that provide a detailed description of the gait of each animal. In order to reduce dimensionality in these datasets we used a multivariate statistical analysis, principal component analysis (PCA; [29]) using the online software ClustVis [30]. The first two principal components were enough to account for about 50% of the total variance of the studied samples, demonstrating the high correlation between the variables describing gait. To visualize the differences between Sema7A deficient and WT mice, we plotted coordinates from each mouse in the new space created by the first two principal components (Figs. 2a, 5a). We then measured the average scores of PC1 and PC2 over a 21 days recovery period. The analysis of factors loadings illustrates the correlations between distinct aspects of locomotion and principal components (Fig. 5a; ESM_6). In this representation, variables that correlate positively or negatively with a given principal component identified clusters of parameters that account for a specific difference between genotypes (red colors indicate higher positive or negative correlation). Out of the factor loadings we identified parameters (here for example, hindlimb print area) that are selectively affected in Sema7A deficient mice.

### Tissue processing and histology

Mice were deeply anesthetized and perfused transcardially with PBS/Heparin (Braun) followed by 4% paraformaldehyde (PFA) in 0.1% PB. The tissue was post-fixed overnight in 4% PFA at 4 °C followed by dissection of brain and spinal cord and cryoprotection in 30% sucrose (Sigma) for at least 48 h at 4 °C.

#### Visualization of serotonergic fibers and cells (5-HT), serotonin receptor 2α (5HTR2α) and cholin acetyltransferase^+^ (ChAT^+^) motoneurons

Staining for motoneurons, serotonergic fibers and the 2α receptor was done sequentially. First 40 µm coronal (transverse) sections were incubated overnight at 4 °C with a goat-α-ChAT (1:100, Merck Millipore AB144P) primary antibody and secondary antibody staining was performed with a donkey-α-goat Alexa488 for 2 h at room temperature (RT; 1:500, Thermo Fisher Scientific, A11055). Consecutively, sections were stained with rabbit-α-5HT overnight at 4 °C (1:10,000, Immunostar 20080) followed by incubation with donkey-α-rabbit HRP (1:100, Thermo Fisher Scientific, A16035) for 1 h at RT and then for 5 h with tyramide coupled to Alexa594 at RT (1:100, Thermo Fisher Scientific Tyramide kit #15). For 5HTR2α staining sections were stained for ChAT as described above, then incubated with rabbit-α-5HTR2a (1:333, Immunostar 24288) for two nights at 4 °C followed by tyramide amplification as described above for the 5HT antibody.

#### Visualization of hindlimb corticospinal tract (hCST) collaterals and contacts on LPSN

To stain hindlimb CST collaterals entering the grey matter at the cervical spinal cord level we cut 50 µm coronal (transverse) sections of the cervical spinal cord (C3–C5) of mice that had a dorsal thoracic hemisection, LPSN labelling and BDA injected in the motor cortex. All sections were first incubated in ABC complex (Vector Laboratories) over night at 4 °C, then washed and incubated with tyramide (Biotin-XX, TSA kit #21, Thermo Fisher Scientific T20931) for 30 min at RT, washed again and incubated with Streptavidin-FITC or -Alexa488 for 2 h at RT (1:500, Thermo Fisher Scientific, SA10002 and S32354). To quantify CST regenerative sprouting at the lesion site, longitudinal consecutive sections from the lesion site were cut (40 µm) and stained as described above.

#### Visualization of layer V motoneurons

Brains with backtraced layer V motoneurons in the cortex were sectioned coronally (frontal plane) consecutively at 40 µm and stained overnight at 4 °C with mouse-α-NeuN (1:500, neuronal nuclei antibody, Merck Millipore MAB377). For antibody detection goat-α-mouse Alexa488 incubated for 2 h at RT (1:500, Thermo Fisher Scientific) was used and nuclei were counterstained with Neurotrace 435 (1:500, NT435, Thermo Fisher Scientific N21479).

#### Lesion site staining

To visualize the lesion site, 40 µm longitudinal sections approximately from T6–L2 were stained with NT435 (1:500, NT435, Thermo Fisher Scientific N21479) or rat-α-GFAP incubated overnight at 4 °C (1:500, Thermo Fisher Scientific 13-0300) followed by goat-α-rat Alexa633 incubated overnight at 4 °C (1:500, Thermo Fisher Scientific A-21094).

#### Visualization of activated astrocytes

For GFAP staining, spinal cords were dissected and post-fixed before they were transferred into 30% sucrose. Longitudinal 40 µm sections of the spinal cord containing the lesion area and surrounding unlesioned tissue were cut and stained for GFAP (glial fibrillary acidic protein). For this purpose, sections were incubated for two nights at 4 °C in rat-α-GFAP, then stained overnight with goat-α-rat Alexa 488 at 4 °C (1:500, Thermo Fisher Scientific A11006) secondary antibody, followed by a 5 min DAPI (1:10,000, Sigma) staining at RT to visualize nuclei.

#### Visualization of macrophages, microglia and lymphocytes

Longitudinal spinal cord sections (40 µm) from T6–T12 spanning the lesion site were stained overnight at 4 °C with rat-α-CD45 (1:250 BD Biosciences 550539) and rabbit-α-Iba1 (1:1000, SYSY, 234013) followed by goat-α-rat Alexa 488 and goat-α-rabbit 594 incubated for 1 h at RT. Nuclei were stained 5 min with DAPI at RT.

#### Visualization of synapsin-positive boutons onto 5HT collaterals

Coronal (transverse) sections (40 µm) of the lumbar spinal cord (L1–L4) were stained with rabbit-α-5HT (1:500, Immunostar 20080) and chicken-α-synapsin 1/2 (1:200, SYSY, 106 006) overnight at 4 °C followed by goat-α-rabbit Alexa 594 and goat-α-chicken 647 for 1 h at RT (1:500, Thermo Fisher Scientific, A-11012 and A-21472).

### Imaging

All confocal images were acquired with a Leica TCS SP8 confocal (Leica Microsystems) with 20 ×, 40 × or 63 × oil immersion objectives and appropriate laser lines and filter sets and no zoom if not otherwise stated.

### Quantifications

All quantifications were performed by an investigator blind to the genotype of the animal except for the analysis of FACS data.

#### Quantification of serotonergic fibers, cells and contacts on motoneurons

**Fig. 1:**
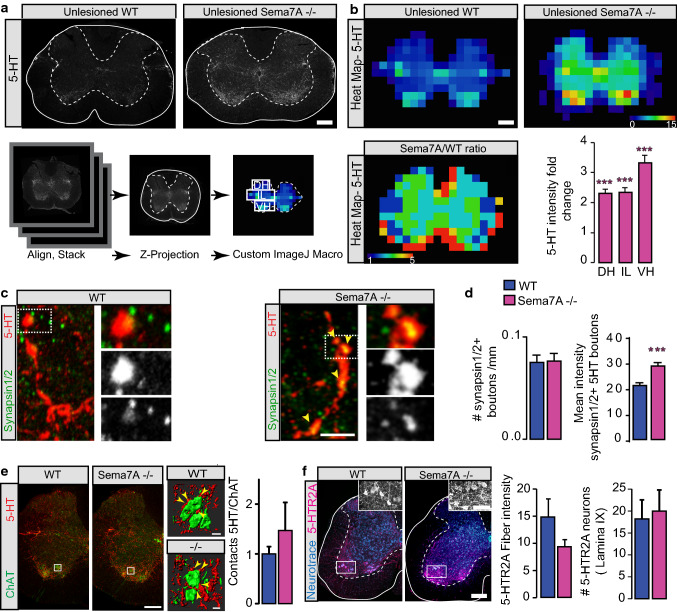
Excessive innervation and altered topography of descending serotonergic connections in the spinal cord of Sema7A deficient mice. **a** Analysis of serotonergic projections in Sema7A deficient and WT mice. Top panel: representative confocal images of serotonergic projection in Sema7A deficient and WT mice. Bottom panel: scheme of the analysis to create heatmaps from several aligned and stacked confocal sections of the L1 spinal cord that are Z projected. Heatmaps were created with custom-written macros in Fiji. Outlined in the heatmap in (**a**) are the three areas used for the quantifications obtained in (**b**) e.g. dorsal horn (DH), intermediate laminae (IL) and ventral horn (VH). **b** Heatmaps of serotonergic projections in Sema7A deficient and WT mice (top) and heatmaps of the fold change ratio of serotonergic projections intensities between Sema7A deficient and WT mice (*n* = 3 per each group). Quantification of relative 5-HT fold change in the dorsal horn (DH), intermediate laminae (IL) and ventral horn (VH). Heatmaps go from dark blue (very low fiber intensity) to bright red (very high fiber intensity). Two-way ANOVA with Bonferroni multiple comparison post-hoc test *p* values: DH: 0.0003; IL: 0.0001; VH: < 0.0001. **c** Confocal images of 5-HT boutons doubled-stained with synapsin 1/2 in the spinal cord of unlesioned WT mice (left) and unlesioned Sema7A deficient mice (right). **d** Quantification of the density of synapsin-positive 5-HT boutons in WT and Sema7A deficient mice (left) and quantification of the mean intensity of synapsin 1/2 positive 5-HT boutons in WT and Sema7A deficient mice (right). Mann–Whitney test: *p* value _(synapsin bouton density)_ = 0.8343; *p* value _(mean intensity of synapsin1 positive 5HT boutons)_ < 0.0001; *n* = 120 boutons analyzed (left) *n* = 372 boutons analyzed (right). **e** Confocal images of putative contacts between serotonergic terminals and motoneurons in the lumbar cord of Sema7A deficient and WT mice and 3D reconstruction of the contacts using Imaris software (yellow arrowheads indicate putative sites of contacts). Quantification of the number of contacts between serotonergic terminals (red) and motoneurons labeled with Choline acetyltransferase (ChAT; green) (*n* = 3 per group; Welch’s test *p* value: 0.4937). **f** Confocal pictures of 5-HT receptors 2α expression in the spinal cord of Sema7A deficient and WT mice. Boxed areas on the pictures are magnified and quantifications of the intensity of 5-HTR2α staining and of the number of neurons expressing 5-HTR2α are presented (WT: blue bars and Sema7A deficient: pink bars; *n* = 3 per group; Welch’s test *p* value: 0.4607). Scale bars equal 200 µm in (**a**, **b**, **e**, **f**), 100 µm in (**f**; insets), 20 µm in (**e**; right panel) and 5 µm in (**c**)

For quantification of 5-HT intensity around the lesion site we used the integrated density function in Fiji and normalized to a clearly negative region of the cord. To generate heat maps of the lumbar area in Fiji a custom written script was used. In brief, we used maximum intensity projections of all confocal scans taken with identical scan settings and aligned them to the central canal for all animals of one genotype (three sections per animal, three animals per genotype and time point). Then an average intensity projection was calculated and a heatmap was generated by averaging the intensity within 200 squares over the images. The intensity was normalized to the intensity within the dorsal funiculus that does not contain 5-HT^+^ fibers. We then created Sema7A/WT ratio by dividing for each square the Sema7A−/− map by the mean (per animals) WT map. We then rounded up each square to the closest integer with five as upper range. Those Sema7A/WT ratio are presented in Figs. 1 and 3. Quantifications were then performed over representative areas of the dorsal horn (DH), ventral horn (VH) or intermediate laminae (IL) as represented in the figures. To quantify the number of serotonergic cells that project to the spinal cord we imaged 5-HT stained brainstems at − 6.36 mm, − 6.96 mm, − 7.48 mm and − 8.00 mm from bregma with a Leica DM4 and the SI software (1 plane in Z). Images were then stitched and cells in the raphe obscurus nucleus (Rob) that project to the spinal cord ventral horn and intermediate grey matter respectively were counted. To quantify contacts of 5-HT fibers on ChAT^+^ cells in the lumbar spinal cord we performed 3D reconstructions of confocal scans using Imaris software (Bitplane) (40 µm, 20 ×, *Z* step size: 1.042 µm).

#### Quantification of synapsin-positive 5-HT collaterals into the lumbar cord

First, to determine the length of the 5-HT collaterals and the number of bouton per μm collateral, images of the ventral horn of lumbar spinal cord sections (40 μm thickness, sections randomly taken, 63 × magnification, *Z* step size 0.4 µm) were acquired (five sections per animal, three animals per genotype and time points). Image stacks obtained with confocal microscopy were processed using Fiji software. The length of all individual collaterals in those sections were measured within the stack with the help of the measurement tool of Fiji (Simple Neurite Tracer). Boutons on 5-HT collaterals were counted (40–70 collaterals per group were analyzed). A bouton was defined as a thick varicosity along a comparably thin 5-HT collateral in the lumbar spinal cord (at least twice bigger and brighter than the adjacent collateral and rounded shape [31]). The total number of boutons per animal was divided by the total length in micrometers of collaterals per animal to calculate the density in boutons/μm. Then, to determine the proportion of boutons that express the synaptic vesicle‐associated protein, synapsin 1/2, sections were stained with anti‐synapsin 1/2 antibody as described above. Image stacks were used for analysis, and maximum intensity projections were generated for figure representation. The percentages of synapsin 1/2‐positive boutons were determined in confocal image stacks (single planes from the image stacks were analyzed) upon the following criteria: boutons were identified as defined above and such boutons were considered synapsin 1/2 positive when the synapsin 1/2 mean intensity within the bouton was twice brighter than the mean intensity background and when the synapsin signal covered the bouton but did not extend beyond it. The number of boutons positive for synapsin 1/2 was expressed as a percentage of all 5-HT boutons by the total length in micrometers of collaterals. The mean intensity for each synapsin 1/2-positive 5-HT boutons was also reported. A minimum of 100 boutons per group was analyzed.

#### Quantification of hCST fibers entering the cervical spinal cord

To analyze hCST fibers entering in the cervical spinal cord, fibers were stained as described above and confocal scans (50 µm, *Z* step size 1.042 µm) of 20 sections were acquired. All images were then processed in Fiji (https://fiji.sc/) to generate maximum intensity projections. Length tracing, counting of total collaterals and exiting collaterals, branchpoints and boutons were also done in Fiji and the NeuronJ plugin [32] was used for length quantification of the total CST grey matter collaterals. To account for differences in CST labelling between the animals all fibers in the tract were counted in the dorsal funiculus at cervical levels in three sections at cervical levels C3 and the length as well as the number of collaterals was normalized to this number. A bouton was counted, if a thick varicosity was clearly visible along an axon. All bouton numbers were normalized to the total length of collaterals for each animal and plotted per µm (as previously established in [23–25, 33]. To analyze CST fiber distribution in the cervical and lumbar spinal cord all images were aligned on the central canal and a grid was placed in Fiji. Using the NeuronJ plugin all collaterals in one area were traced and the distribution of fibers in the quadrants was calculated.

#### Quantification of hCST contacts on LPSN

Contacts between the hCST and LPSN were counted using a fluorescent microscope (Olympus IX71) with a 40 ×/0.65 air objective. We counted the total number of contacts and the total number of LPSN contacted by labelled CST collaterals. A contact was defined as a close apposition between a CST bouton and a LPSN. All numbers were normalized to the number of TexasRed^®^ labelled neurons and the number of labelled CST fibers in the white matter tract.

#### Collateral sprouting at the lesion site

To quantify collateral sprouting at the lesion site, all lesion sites were imaged with a Leica DM4 (Leica microsystems) fluorescence microscope and the Stereo Investigator Software (SI, MBF) or a Leica TCS SP8. All stacks were then projected and stitched in Fiji and a 200 µm grid was placed on the stitched image, the 0 line representing the location of the CST stump. The number of sprouts on longitudinal sections (average of 8.6 sections per animals) crossing each line was counted and normalized to the number of CST fibers labelled in the white matter and the number of sections counted.

#### Quantification of the number of layer V traced motoneurons in the brain

For quantification of TexasRed^®+^ motoneurons in the brain we counted all labelled cells in every second section of the brain from the first section to the last section containing labelled neurons on an Olympus IX70 with a 10 × air objective. All results are mentioned as mean per section counted.

#### Quantification of lesion volume

To measure the lesion volume every second section of the lesion area stained with NT435 was imaged with an Olympus IX71 with a 4 × objective. The lesion was outlined and measured in Fiji and values were multiplied by 80 µm and summed up to calculate the total lesion volume.

#### Quantification of reactive astrocytes

For GFAP quantification three 40 × stacks (40 × magnification, 0.502 µm step size, 20 µm central part of the stack analyzed) of each region of interest (lesion, lesion border, a randomly chosen area outside of the lesion) were acquired with a Leica TCS SP8 confocal by an investigator blinded to the genotype of the investigated animal. A cell was deemed as GFAP^+^ if at least 2/3 of the DAPI^+^ nucleus is encased by GFAP staining.

#### Quantification of CD45 and Iba1 positive cells in and around the lesion

For cell quantification 5 µm longitudinal stacks were maximum intensity projected and masks outlining the lesion site, the lesion border and unaffected tissue were generated using Fiji (https://fiji.sc/). Automatic cell counting was done using CellProfiler software [34] using the generated masks for area discrimination. As a first step all DAPI positive nuclei were outlined and counted, followed by automatic detection of CD45 and Iba1 positive cells. A positive cell was only counted if it overlaid a DAPI positive nucleus. Results were calculated per area.

### Image processing

All images were stitched and maximum intensity projected either in LasX (Leica microsystems) or Fiji. For illustration images were processed in Adobe Photoshop using gamma adjustment to enhance visibility if necessary. Gamma adjustments were used in images representing intensity differences e.g. in serotonergic innervation for visualization purposes only.

### Flow cytometry (FACS)

For analysis of immune cell populations around the lesion site, mice were sacrificed, spinal cord from approximately T6–L2 isolated and transferred to ice-cold PBS (Sigma-Aldrich). Tissue was cut in small pieces and digested in RPMI containing 2% fetal calf serum (Sigma-Aldrich), 25 mm HEPES (Sigma-Aldrich), DNase I (10 ng/ml, StemCell Technologies) and Collagenase D (0.8 mg/ml, Roche) for 30′ at 37 °C. Reaction was stopped by adding 1:100 dilution of a 0.5 M EDTA (Sigma-Aldrich) solution. Suspension was filtered through 70 µm cell strainers (Falcon) and re-suspended in a 30% solution of Percoll (Sigma-Aldrich). After 30′ of gradient centrifugation at 10.800 r.p.m., the top (myelin) and lower (red cells) layers were removed and the remaining solution was filtered through 70 µm cell strainers (Falcon). Stainings were performed in ice-cold PBS after Fc-receptor blockade (CD16/32, 1 µl/million cells, clone 2.4G2, BD Biosciences) using LIVE/DEAD staining (1:100, Invitrogen) and the following antibodies: CD45 (1:400, clone 30-F11, BioLegend), CD11b (1:300, clone M1/70, BD Biosciences), Ly6C (1:200, clone AL21, BD Biosciences), Ly6G (1:200, clone 1A8, Biolegend), CD4 (1:200, clone RM4-5, BioLegend). Samples were acquired on a LSR-Fortessa cytometer (BD Biosciences) and results analyzed by FlowJo software.

### Statistical evaluation

All results are given in mean ± SEM. For statistical evaluation, we used GraphPad Prism 7.01 (GraphPad, USA). We first tested for normality and then used one- and two-ways ANOVA with Bonferroni post-hoc to test for significance in the behaviour data. For two columns comparison between the genotypes, we first tested for normality and then used the appropriate statistical test e.g. an unpaired *t* test for normally distributed data sets and a Mann–Whitney test for data sets that did not distribute normally. Significance levels are indicated as follows: *p < 0.05; **p < 0.01; ***p < 0.001.

## Results

### Excessive innervation and altered topography of descending serotonergic connections in the spinal cord of Sema7A deficient mice

To determine the influence of Sema7A on the development of the spinal serotonergic innervation, we first investigated the pattern of spinal cord 5-HT projections in the lumbar spinal cord. We observed that Sema7A deficient animals showed an overall increased density of 5-HT profiles in the lumbar spinal cord (Fig. 1a; 696 ± 149 vs 322 ± 84 AU). Serial counting of 5-HT neurons in the raphe obscurus of Sema7A deficient and WT mice did not reveal any significant difference in the total number of 5-HT neurons from which these fibers originate (ESM_1a, b) indicating that the excessive innervation observed in Sema7A deficient mice is likely due to increase branching and growth of descending 5-HT axons. We then created heatmaps to better define the topographical distribution of enhanced 5-HT innervation in Sema7A deficient mice (Fig. 1a). Quantification of 5-HT axonal density in dorsal, intermediate and ventral regions suggests that 5-HT profiles are significantly increased in all regions of the lumbar spinal cord (Fig. 1b). To analyze the integration of those 5-HT boutons and determine whether this increased serotonergic innervation of the spinal cord results in excessive contacts onto the neurons that execute motor behaviour i.e. motoneurons, we focused on two additional analysis in the ventral horn. First we determined whether the density of boutons expressing synapsin 1/2 onto 5-HT collateral and the intensity of synapsin 1/2 into those boutons would be affected by the mice genotype (WT vs Sema7A deficient). While we saw no differences in the number of synapsin 1/2+ boutons, we observed that the mean intensity of synapsin 1/2 (corrected for the size of the boutons) was higher in Sema7A deficient mice compared to WT mice (Fig. 1c, d). Second, we focused on contacts onto motoneurons as it is well-known that serotonergic projections in the spinal cord initiate rhythmic locomotor activity by acting on these neurons [9, 35, 36]. To do so, we stained for choline acetyltransferase (ChAT) and analyzed appositions between 5-HT profiles and ChAT positive motoneurons. We found no significant changes in the number of contacts between 5-HT axons and ChAT positive motoneurons in WT compared to Sema7A−/− mice (Fig. 1e). These data indicate that homeostatic mechanisms [37–39] during development can maintain pre-set motoneuron contact patterns even in the presence of an excessive number of serotonergic fibers. To more systematically understand how the increased presence of serotonergic fibers impacts synaptic connections we studied the density of 5HT2α receptors, which are the major serotonin receptors in the spinal cord expressed in particular on ventral motoneurons [40–43]. Our results show that, in contrast to 5-HT fibers, 5-HT2R2α expression is similar in control and Sema7A deficient animals (Fig. 1f) again supporting the concept that homeostatic regulation preserves similar levels of serotonergic transmission despite a marked increase in serotonergic innervation.

Finally, we wanted to examine whether the changes of the serotonergic projections were specific to this tract system or a sign of a more general alteration of the brain—spinal cord connectivity. To do so we studied the anatomy of the corticospinal tract system another major descending motor tract that expresses PlexinC1 [22] and that regulates skilled aspects of locomotion as well as fine motor control [23, 44]. We therefore traced the hindlimb portion of the corticospinal tract by stereotactic injection of the anterograde tracer biotinylated dextran amine (BDA) and investigated whether Sema7A−/− would show aberrant CST projections. As anticipated we could detect almost no hindlimb CST fibers in the cervical spinal cord of both Sema7A−/− and WT mice while a dense CST innervation of the lumbar spinal cord was found in both genotypes. However neither the density nor the topographical organisation of lumbar CST collaterals were different in Sema7A-deficient and Sema7A-compentent mice (ESM_2) indicating that alteration of the serotonergic projection patterns are not a feature of a generally altered connectivity of the lumbar spinal cord. Retrogradely traced CST upper motoneurons in the cortex were also similar in number and location (WT 31.29 ± 6.64; Sema7A−/− 22.52 ± 6.59).

### No functional changes in healthy Sema7A deficient mice

**Fig. 2:**
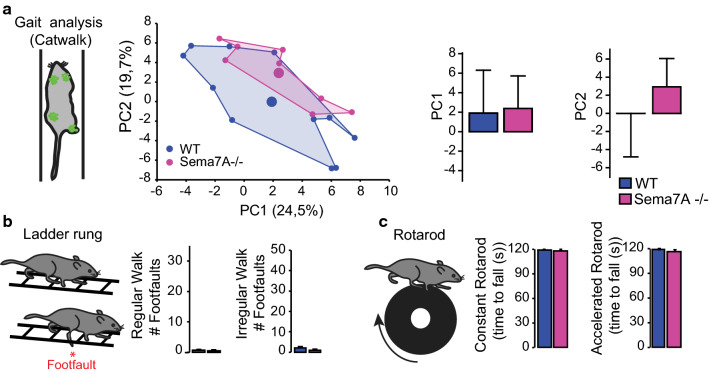
Altered serotonergic innervation patterns are functionally compensated in healthy Sema7A deficient mice. **a** Gait analysis of Sema7A deficient and WT mice using the Catwalk^®^ (scheme on the left). Middle: two-dimensional statistical representation of gait parameters after performing principal component analysis (PCA). The area defined by individual points (blue: WT and pink: Sema7A deficient) is traced to emphasize the gait patterns of each genotype which are largely overlapping (KO: *n* = 8; WT: *n* = 11 mice). Right: Bar graphs of average scores on principal components 1 and 2 (PC1 *p* value = 0.9039; PC2 *p* value = 0.1774; Mann–Whitney *U* test). **b** Quantification of the functional performance of Sema7A deficient (pink lines, *n* = 15), and WT mice (blue lines; *n* = 12) in the ladder rung test (scheme left panel, regular walk middle panel; *p* value = 0.5750; irregular walk, right panel; *p* value = 0.08326 two-way ANOVA with Bonferroni multiple comparison post-hoc test). **c** Quantification of the functional performance of Sema7A deficient (pink lines, *n* = 14), and WT mice (blue lines; *n* = 14) in the rotarod test (scheme left panel) (constant speed middle panel; *p* value = 0.5498; accelerated speed, right panel; *p* value = 0.8402; two-way ANOVA with Bonferroni multiple comparison post-hoc test)

We then asked whether the excessive and topographically altered innervation by 5-HT fibers in the lumbar spinal cord can trigger perturbations in locomotion or whether those changes can be functionally compensated. We therefore analyzed gait and locomotor abilities of Sema7A deficient animals using several behavioural tests that investigate gait (Catwalk^®^, [28]), rhythmic and skilled walking (ladder rung test, [26]) and locomotor coordination and balance (rotarod test, [45]). Our results show that in the mature intact spinal cord, the over-innervation of serotonergic fibers did not lead to significant changes in gait abilities (Fig. 2a), the ability to perform either rhythmic or coordinated paw placement (Fig. 2b) or in balance and motor endurance (Fig. 2c). These findings further support the notion that when Sema7A expression is constitutively deleted compensatory strategies emerge to maintain locomotor function.

### Adaptive remodeling of serotonergic connections in response to spinal cord injury is disturbed in Sema7A deficient mice

**Fig. 3:**
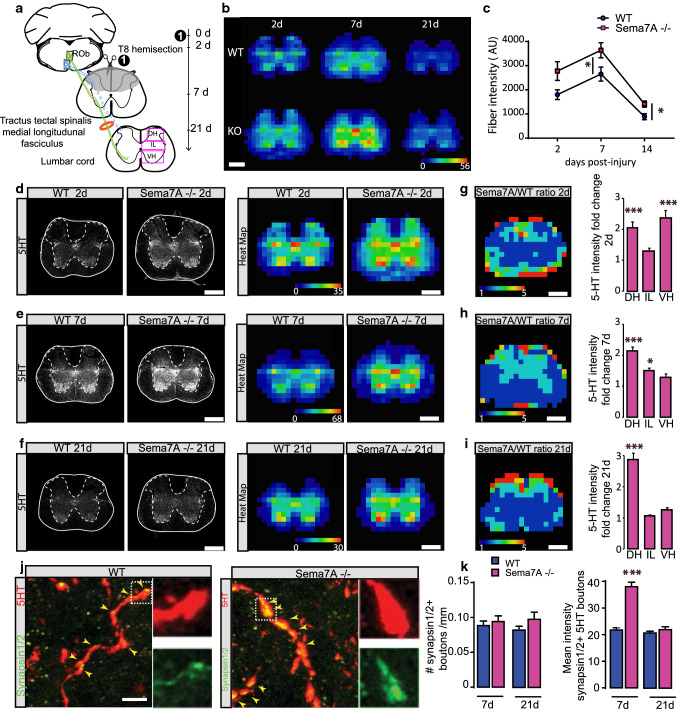
Adaptive remodeling of serotonergic connections in response to spinal cord injury is disturbed in Sema7A deficient mice. **a** Left: scheme of the descending serotonergic axis studied. We focused on descending ventral 5-HT projections from the Tractus tectal spinalis medial longitudinal fasciculus that is not interrupted by the T8 dorsal hemisection in mice. Note that the substantia gelatinosa tract is cut from the lesion. Outlined in the lumbar cord representation are the three areas used for the quantifications obtained in (**g**, **h**, **i**) e.g. dorsal horn (DH), intermediate laminae (IL) and ventral horn (VH). Right: timeline of the experiment following spinal cord injury. **b** Heatmaps of 5-HT fibers intensity in the lumbar cord at L1 Sema7A deficient and WT mice at different time points following spinal cord injury. Heatmaps go from dark blue (very low fiber intensity) to bright red (very high fiber intensity). **c** Quantification of relative 5-HT intensity at different time points following spinal cord injury (blue lines: WT mice; pink bars: Sema7A deficient mice; *n* = 3 for all groups; *p* values: 2 days = 0.0499; 7 days = 0.0266, 21 days = 0.4431). **d**–**f** Confocal images and heatmaps of overall relative 5-HT intensity at 2 days (**d**), 7 days (**e**) and 21 days (**f**) at the lumbar L1 segment of the spinal cord following spinal cord injury. **g**–**i** Heatmaps of the fold change ratio of serotonergic projections intensities between Sema7A deficient and WT mice and quantification of relative 5-HT fold change in the dorsal horn (DH), intermediate laminae (IL) and ventral horn (VH) at 2 days (**g**), 7 days (**h**) and 21 days (**i**) (*p* values: 2 days: DH = 0.0042; IL = 0.9839: VH = 0.002; 7 days: DH ≤ 0.001; IL = 0.0127: VH = 0.091; 21 days: DH ≤ 0.001; IL ≥ 0.9999: VH ≥ 0.9999). Heatmaps go from dark blue (very low fiber intensity) to bright red (very high fiber intensity). **j** Confocal images of 5-HT boutons doubled-stained with synapsin 1/2 in the spinal cord of WT mice (left) and unlesioned Sema7A deficient mice (right) 7 days following spinal cord injury. Insets are magnified three times. **k** Quantification of the density of synapsin-positive 5-HT boutons in WT and Sema7A deficient mice (left) and quantification of the mean intensity of synapsin 1/2 positive 5-HT boutons in WT and Sema7A deficient mice (right) at 7 days and 21 days following spinal cord injury. Mann–Whitney test: *p* value_(synapsin bouton density 7 days)_ = 0.7558; *p* value_(synapsin bouton density 21 days)_ = 0.1869; *p* value _(mean intensity of synapsin1 positive 5HT boutons 7 days)_ < 0.0001; *p* value _(mean intensity of synapsin1 positive 5HT boutons 21 days)_ = 0.2743; *n* = 103–108 boutons analyzed (left); *n* = 191–364 boutons analyzed (right). Scale bars in (**b**, **d**–**i**) equal 400 μm. Scale bar in **j** equals 5 µm. Panels **c**, **g**–**i**: two-way ANOVA with Bonferroni multiple comparison post-hoc test. Panel **k**: Mann–Whitney tests at 7 and 21 days

**Fig. 4:**
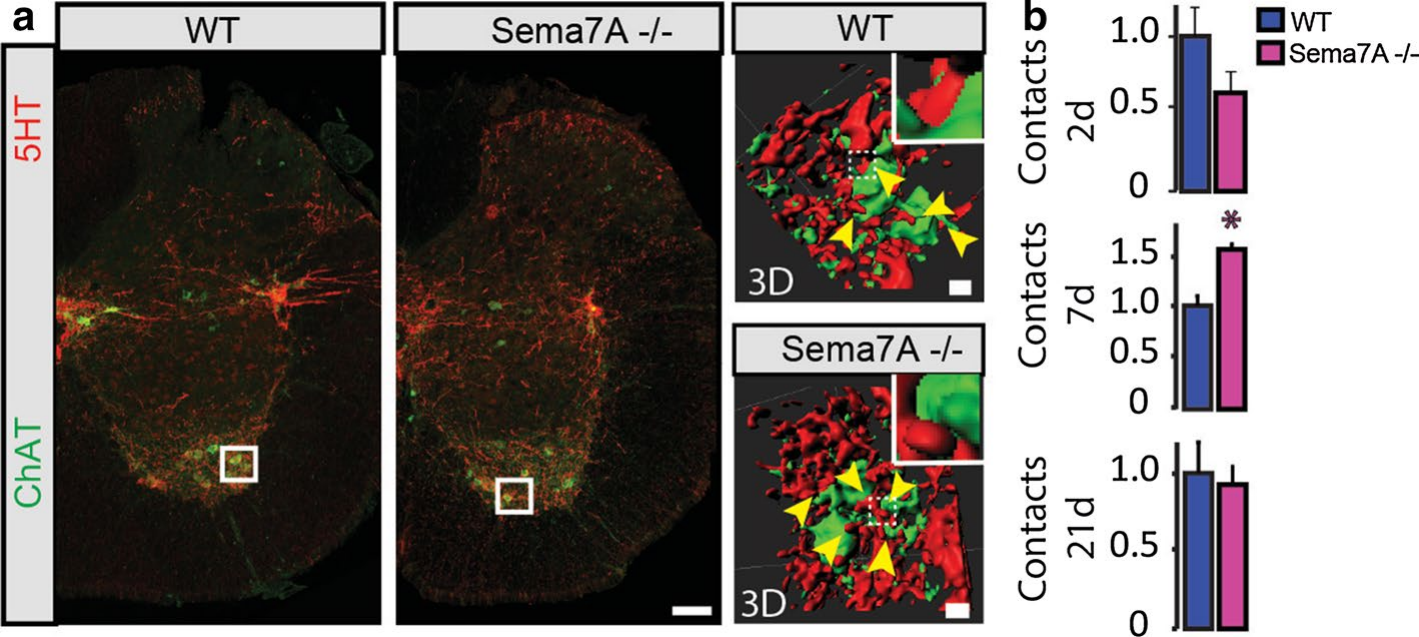
Deletion of Sema7A triggers only transient changes in the contact formation rate onto motoneurons following spinal cord injury. **a** Confocal images of putative contacts between serotonergic terminals and motoneurons in the lumbar cord of Sema7A deficient and WT mice and 3D reconstruction of the contacts using Imaris software (yellow arrowheads indicate putative sites of contacts and insets represent areas of contact). **b** Quantification of the number of contacts between serotonergic terminals (red) and motoneurons labeled with Choline acetyltransferase (ChAT; green) (*n* = 3 per group, except at 2 days *n*_WT_ = 4; *p* values: 2 days = 0.5318; 7 days = 0.0134; 21 days = 0.7723; all Welch’s *T* test). Scale bars equals 100 µm and 20 µm in the Imaris reconstruction in (**a**)

The serotonergic control of spinal locomotor networks not only play an important role for the generation of rhythmic locomotion in healthy mice but are also critical for the re-establishment of such movement capabilities after injury [29]. In order to examine whether Sema7A is also an important molecular cue during the remodeling of serotonergic connections after injury, we introduced an incomplete spinal cord hemisection at thoracic spinal level 8 that interrupted the descending 5-HT input to the spinal cord leaving the serotonergic fibers in the tractus tectal spinalis and the medial longitudinal fasciculus intact [46] (Fig. 3a). As we previously showed that the expression of Sema7A distal to the lesion site in the cord is not altered following spinal cord injury [22], we then proceeded to map regional intensities of 5-HT profiles at 2, 7 and 21 days following the spinal cord injury and represented them as heatmaps (as described above). We found that following the spinal cord injury the serotonergic innervation of the spinal cord was increased at the initial time point (2 days) after injury in line with the altered innervation status in healthy mice and remained significantly higher than in wildtype mice over the subsequent remodeling period (7 and 21 days post-injury, Fig. 3b, c). Interestingly, the serotonergic innervation levels in both wildtype and Sema7A deficient mice evolved in parallel over time and after an initial increase at 7 days after injury then decreased up to 21 days (Fig. 3c). We then examined the topographic organisation of the remodeling 5-HT fibers in the dorsal horn, intermediate laminae and ventral horn of the spinal cord (Fig. 3a). Here we found that over time an unbalance of the serotonergic innervation pattern developed in Sema7A-deficient mice: while the density of 5-HT profiles in the intermediate layers and the ventral horn of the spinal cord over time approached similar levels in wildtype and Sema7A deficient mice, serotonergic innervation of the dorsal horns was nearly threefold increased in Sema7A deficient mice compared to wildtype mice at the end of the remodeling period at 21 days after injury (Fig. 3d–i). To determine whether this unbalance of serotonergic innervation was coupled with changes of synapse formation along the fibers we probed for boutons along the 5-HT fibers, in particular using double-labeling with synapsin 1/2. We focused on the 7 days and 21 days time points as the acute time point resembles the uninjured situation. Here we found no changes in synapsin-positive bouton density along the 5-HT collaterals in the ventral horn when both group of animals were compared (Fig. 3j, k). However we observed that the intensity of synapsin within 5-HT boutons was almost doubled in Sema7A deficient mice at 7 days post-injury (Fig. 3k). Those values then reach WT values 21 days post-injury (Fig. 3k). In line with the normalization of the serotonergic innervation pattern in the ventral horn observed over time, we found only transient changes in the number of contacts between 5-HT profiles and motoneurons at 7 days post-injury that normalized at later time points (Fig. 4a, b). Likewise when we analyzed the expression of 5-HT2R2α in the lumbar cord at the different time points following injury, we observed no significant changes neither between WT and Sema7A deficient animals or over time (2 days: WT 14.1 ± 1.9 vs Sema7A−/− 10.0 ± 1.3; 7 days: WT 15.5 ± 1.6 vs Sema7A−/− 12.1 ± 1.7; 21 days WT 9.1 ± 2.2 vs Sema7A−/− 4.8 ± 0.8, *n* = 2–3 per group).

Next we wanted to investigate whether the topographical alterations in post-injury remodeling observed in Sema7A deficient mice are specific to serotonergic connections or a sign of a more general alteration of axonal remodeling processes. We therefore investigated the regeneration and remodeling of the corticospinal tract system. For this purpose we performed a bilateral dorsal hemisection of the spinal cord at T8 in Sema7A-deficient mice and age-matched Sema7A-competent mice and anterogradely traced the hindlimb portion of the CST. Analysis of the lesion site in the spinal cord showed that CST sprouting/regeneration is comparable between Sema7A deficient and competent mice (ESM_3a–c) and analysis of the cervical spinal cord revealed that while aspects of the CST hindlimb fibers entering the grey matter are enhanced (ESM_3d–h), the formation of CST detour circuits, which are important contributors to the recovery of CST function [23, 24, 44] are not altered in Sema7A deficient mice (ESM_3i–k). Furthermore, we did not observe any changes in the topographic organization of the hCST projections to the cervical spinal cord (ESM_4). Taken together these data indicate that Sema7A is an important regulator of serotonergic remodeling in the injured CNS that is required for proper targeting of serotonergic inputs to the lumbar spinal cord.

### Sema7A signalling is required for proper locomotor recovery following spinal cord injury

**Fig. 5:**
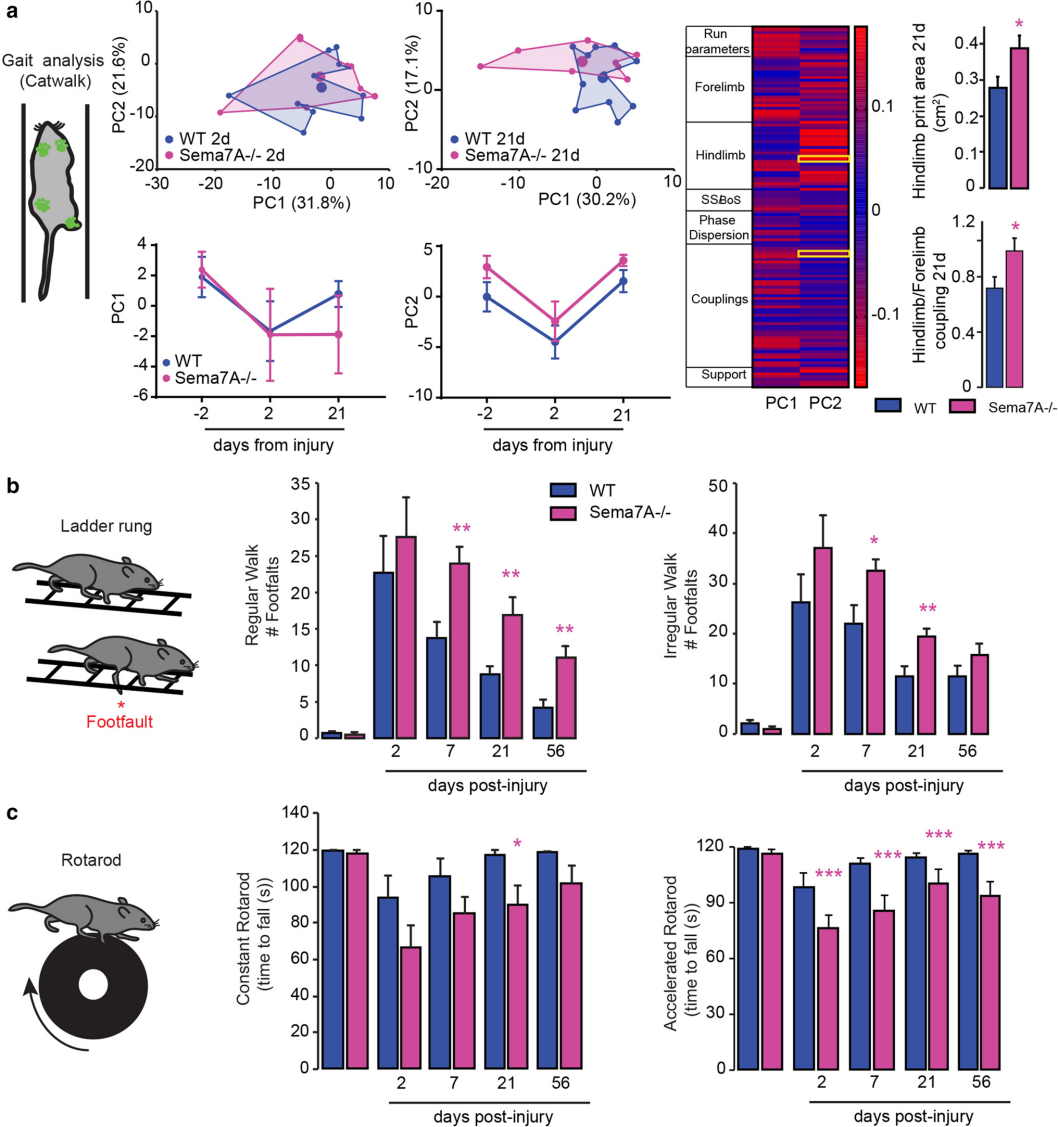
Sema7A signalling is required for proper gait and locomotor recovery following spinal cord injury. **a** Gait analysis of Sema7A deficient and WT mice using the Catwalk® (scheme on the left). Middle left: two-dimensional statistical representation of gait parameters after performing principal component analysis (PCA). The area defined by individual points (blue: WT and pink: Sema7A deficient) is traced to emphasize the specific gait patterns of each genotype at 2 and 21 days post-injury (KO: *n* = 8, WT: *n* = 11 mice). Middle right: overtime evolution of principal components 1 and 2 following spinal cord injury. Right panel: color-coded representation of factor loadings that identify each parameter’s correlation coefficient (*r*) with PC1 and PC2. Parameters with positive/negative correlation are coded in red while those with a correlation close to 0 are coded in blue (see scale). Quantification of hindlimb print area (*p* value = 0.0259) and print position of Hindlimb/Forelimb (*p* value = 0.0203, Mann–Whitney *U* test both) at 21 days extracted from the factor loading. **b** Quantification of the functional performance of Sema7A deficient (pink lines, *n* = 15), and WT mice (blue lines; *n* = 14) in the ladder rung test (scheme left panel, regular walk middle panel; *p* values: baseline = 0.5750, 2 days = 0.5139, 7 days = 0.0036; 21 days = 0.0057, 56 days = 0.0016; irregular walk, right panel; *p* values: baseline = 0.0833, 2 days = 0.2171, 7 days = 0.0200; 21 days = 0.0034, 56 days = 0.1702) following spinal cord injury. **c** Quantification of the functional performance of Sema7A deficient (pink lines, *n* = 14), and WT mice (blue lines; *n* = 14) in the rotarod test following spinal cord injury (scheme left panel, constant speed middle panel; *p* values: baseline = 0.5498, 2 days = 0.13179, 7 days = 0.1362; 21 days = 0.0133, 56 days = 0.0923; accelerated speed, right panel; *p* values: baseline = 0.8402, 2 days ≤ 0.0001, 7 days ≤ 0.0001; 21 days ≤ 0.0001, 56 days ≤ 0.0001). Data are analyzed with a two-way ANOVA with Bonferroni multiple comparison post-hoc test for the ladder rung and the rotarod

Finally, we tried to understand whether the unbalance of serotonergic projections observed in Sema7A deficient mice would influence the functional recovery of the mice following an incomplete spinal cord injury. To assess this, we performed thoracic dorsal bilateral hemisections of the spinal cord and followed the recovery of gait and rhythmic and coordinated locomotor function using behavioural testing paradigms such as the Catwalk^®^ analysis [28], the ladder rung test [26], the rotarod test [47] in Sema7A-deficient mice and Sema7A-competent mice. As the recovery process is likely to be influenced by the lesion extent we first confirmed that the lesion volume is not altered in Sema7A deficient mice (ESM_5a). Furthermore as Sema7A and its receptors can also regulate the glial and immune cell response [48, 49] we performed flow cytometry and immunohistochemical analysis to show that inflammation and reactive gliosis at the lesion site were not changed between the Sema7A deficient and competent mice (ESM_5b–e). Having established this, we then evaluated the recovery of the mice and focused on three parameters: first we evaluated the gait of the animals following the injury, second we analyzed their recovery of balance and endurance (rotarod) and third we assessed the rhythmic stepping and coordinated paw placement (ladder rung). Gait recovery of the mice was analyzed based on the Catwalk^®^ ([28]; Fig. 5a) and using a principal component analysis (PCA) of Catwalk parameters to reduce dimensionality and provide a comprehensive quantification of locomotor features. We visualized gait patterns in the two-dimensional coordinates created by PC1–2, where PC1 and PC2 explains the highest variance (25.4 and 19.7% respectively) and observed that at 2 days following injury, the mean PC values were similar in Sema7A-deficient and WT mice (Fig. 5a). However, over the course of the recovery process the mean PCA values of Sema7A-deficient mice segregated further from that of WT mice suggesting a difference in the gait recovery also this did not reach statistical significance (Fig. 5a). To further understand which parameter are responsible for differential gait recovery, we extracted the distinct characteristics captured by PC1 and PC2 from the analysis of factor loadings and found that a number of rhythmic parameters regulated by spinal locomotor networks including the recovery of the hindlimb position (in this case the hindlimb print area) or the forelimb/hindlimb coupling were significantly altered in Sema7A deficient mice compared to WT mice (Fig. 5a; ESM_6). We then evaluated functional recovery of rhythmic stepping and of coordinated paw placement using the ladder rung test and found that Sema7A deficient mice did not recover stepping abilities as rapidly and precisely as WT mice (Fig. 5b). Interestingly when we compared regular rhythmic walking on the ladder rung, a behaviour controlled by local interneurons-motoneurons adaptations, and coordinated walking on the irregular ladder rung that demands permanent step/length adaptation, a behaviour likely controlled at supraspinal levels, we see that rhythmic behaviour is more perturbed in Sema7A. This is in agreement with the gait analysis showing uncoupling of rhythmic gait movements and most importantly with the loss of patterning of 5-HT input to motoneuronal networks. Similarly, balance and gross locomotion was evaluated using the rotarod and we observed that Sema7A deficient mice showed more pronounced deficits that did not recover over time in a comparable extent as WT mice (Fig. 5c). Finally, we evaluated clasping of the hindlimbs as an early indication of the possible development of a spastic response following spinal cord injury. We could see that Sema7A−/− mice already show impaired clasping at 14 days following injury compared to 21 days for WT mice suggesting an earlier development of a spastic response (ESM_7). Taken together, our results indicate that Sema7A signalling is required for proper remodeling of serotonergic connectivity and timely recovery of locomotion after spinal cord injury.

## Discussion

The proper integration of supraspinal pathways and local spinal circuits is critical for efficient locomotion. In this study we demonstrate for the first time that the presence of Sema7A is critical to the correct patterning of the descending serotonergic projection from the brainstem to the spinal cord. The fact that the patterning of a monoaminergic system can be controlled by Sema7A is not without precedent. Recently Sema7A was shown to control the correct segregation of nigrostriatal and mesolimbic dopaminergic pathways—another monoaminergic system—in the striatum via its interaction with plexinC1 [20]. Whether the effect of Sema7A on the correct targeting of serotonergic projection is also mediated through this plexin receptor or uses integrins that can also transduce Sema7A signaling [15, 16] remains to be determined. However one of our previous study [22] has already shown that not only Sema7A is expressed quite uniformly throughout the different laminae of the adult spinal cord but also its expression pattern appeared to be stable over time and was by large not affected by a thoracic hemisection. Overall our demonstration of exuberant serotonergic innervation in Sema7A deficient mice now indicates that the interaction between Sema7A and its receptor results in a repulsive signal that restricts serotonergic projections to the spinal cord. In this context it is interesting to note that we did not observe marked differences in the number of putative synaptic contacts between 5-HT fibers and motoneurons suggesting that Sema7A, in our system, is not a major regulator of serotonergic synapse formation as has been suggested by recent studies in other axonal populations [18, 19]. As our analysis was performed in adulthood it is of course possible that developmental differences in synapse formation are compensated during later CNS maturation. On the other hand, these data could also indicate that developmental homeostatic mechanisms [37–39] can maintain pre-set motoneuron (more than 90% of them express 5HTR2α) contact patterns and synapse formation even in the presence of excessive serotonergic fibers/pre-synaptic partners. The existence of compensatory strategies that maintain homeostatic function is further highlighted by our observation that unlesioned mice show a normal gait pattern and unaltered locomotor capabilities despite the persistence of excessive serotonergic innervation to the spinal cord. It is indeed known that the control of gait and locomotion is a redundant process that involves several supraspinal inputs from the different monoaminergic systems that project to the spinal cord: serotonergic, noradrenergic and dopaminergic [1]. It is thus conceivable that the adaptation of other monoaminergic projections is induced to balance excessive serotonergic input. In addition to monoaminergic projections other supraspinal tract systems such as the corticospinal tracts are known to be responsible for fine tuning of locomotor control [50, 51]. Our analysis of the corticospinal projections did not reveal any abnormalities indicating that neither does Sema7A directly regulate the patterning of corticospinal projections nor are those projections adapted as part of a compensatory process.

Interestingly when we challenged the system with a spinal cord lesion we observed an altered response pattern of serotonergic fibers in Sema7A deficient mice that indicates that Sema7A also restricts the endogenous remodeling of serotonergic connectivity in adult mice. This is important to note as it indicates—given the fact that semaphorins are expressed in adulthood and following spinal lesions—that the remodeling is not only altered because the circuits are misformed in development but also because Sema7A restricts innervation and patterning of serotonergic fibers during remodeling. In particular, over time, the lack of Sema7A appears to result in an excessive 5-HT projections to the dorsal laminae of the spinal cord. This change in the serotonergic innervation pattern is likely to contribute to the lack of recovery seen following the injury. As maladaptive serotonergic innervation can be responsible for spasticity following spinal cord injury [52–54], it appears likely that the altered pattern of serotonin projections with exuberant projections to the dorsal horn is directly linked to the impaired gait and functional improvements following spinal cord injury. This is shown in our gait data with an altered positioning of the hindpaws in injured Sema7A deficient mice and in our hindlimb clasping test which showed typical signs of spasticity [55]. We also observe that the exuberant serotonin projections are correlated with perturbations in gait and rhythmic locomotion following injury. It is well known that serotonergic neurons located in the medulla initiate rhythmic locomotion activity via direct actions on motoneurons but also indirect actions mediated via spinal interneurons [9, 35, 36] which could be thus perturbed in Sema7A−/− animals. One additional possibility is that afferent input could have changed in the knockout mice contributing to regulation of excessive sprouting of 5-HT. For example, it is known that in the cervical and lumbar spinal cord afferent inputs are progressively retracted postnatally and this is thought to be via competition with descending systems [56, 57]. It could either be that in Sema7A−/− mice, the afferent input is less dense therefore participating to an excessive innervation of 5-HT descending fibers or that the postnatal pruning occurs more efficiently due to the excessive 5-HT innervation. Following injury, Sema7A−/− mice also show unbalanced innervation of 5-HT fibers. Likewise, while afferent sprouting is induced by spinal cord lesions interrupting descending systems, it can be that the existing unbalance of 5-HT sprouting is strengthened and that exuberant sprouting in the dorsal horn in particular is induced in response to denervation of descending systems. Such mechanisms could then be compensated during development but not following lesions in adulthood. In line with other emerging findings [22, 24, 58–60] our study further reinforces the view that the molecules that guide the initial formation of neuronal circuits during development are later reused to instruct the adaptive remodeling of such circuits in the injured mature CNS. Revealing the post-injury actions of these classical developmental molecules will thus be important not only to achieve a better understanding of the mechanistic regulation of remodeling processes but also to develop more refined therapies that translate this knowledge into a targeted support of neuronal plasticity in the injured nervous system. The identification of Sema7A as an important regulator of the serotonergic control of locomotor circuits provides a first step in this direction.

## Electronic supplementary material

Below is the link to the electronic supplementary material.

Supplementary file1 (PDF 1473 kb)

## Data Availability

The datasets generated during the current study are available from the corresponding author on reasonable request.
