# Supplementary material for: Semaphorin 7A restricts serotonergic innervation and ensures recovery after spinal cord injury
Source: Cell Mol Life Sci. 2020 Oct 30;78(6):2911–27. doi: 10.1007/s00018-020-03682-w (PMC8004489; doi:10.1007/s00018-020-03682-w)
Supplement: Supplementary file 1 — Supplementary file1 (PDF 1473 kb) [file 18_2020_3682_MOESM1_ESM.pdf]

ELECTRONIC SUPPLEMENTARY MATERIAL

Semaphorin 7A restricts serotonergic innervation and ensures recovery after spinal cord injury.

Kristina Loy[1,2,3], Julie Fourneau[1,2], Ning Meng[1,2], Carmen Denecke[1,2,3], Giuseppe Locatelli[1,2] and Florence M Bareyre[1,2,4]

1 Institute of Clinical Neuroimmunology, University Hospital, LMU Munich, 81377 Munich, Germany
2 Biomedical Center Munich (BMC), Faculty of Medicine, LMU Munich, 82152 Planegg-Martinsried, Germany
3 Graduate School of Systemic Neurosciences, LMU Munich, 82152 Planegg-Martinsried, Germany
4 Munich Cluster of Systems Neurology (SyNergy), 81377 Munich, Germany

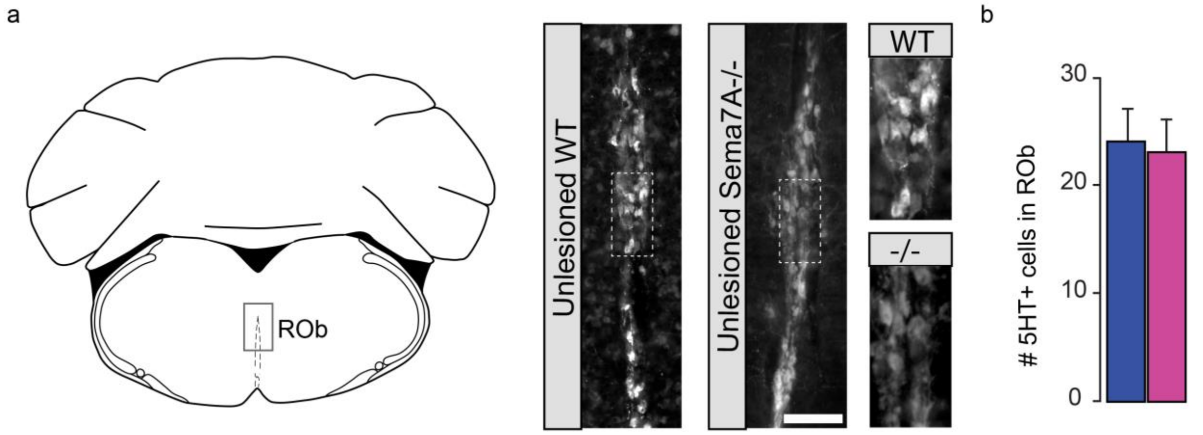

ESM_1 Number of serotonergic upper motoneurons in the Raphe nucleus is not changed in Sema7A deficient mice

a Schematic representation of the Raphe Obscurus area from which descending serotonergic connections are issued and confocal images of the Raphe obscurus in WT (left) and Sema7A deficient (right) mice. Boxed areas are magnified twice on the right.

b Quantification of the number of 5-HT upper neurons in the Raphe obscurus in WT and Sema7A deficient mice (p-value=0,2286 Mann-Whitney-U-Test).

Scale bar equals 100µm in (**a**).

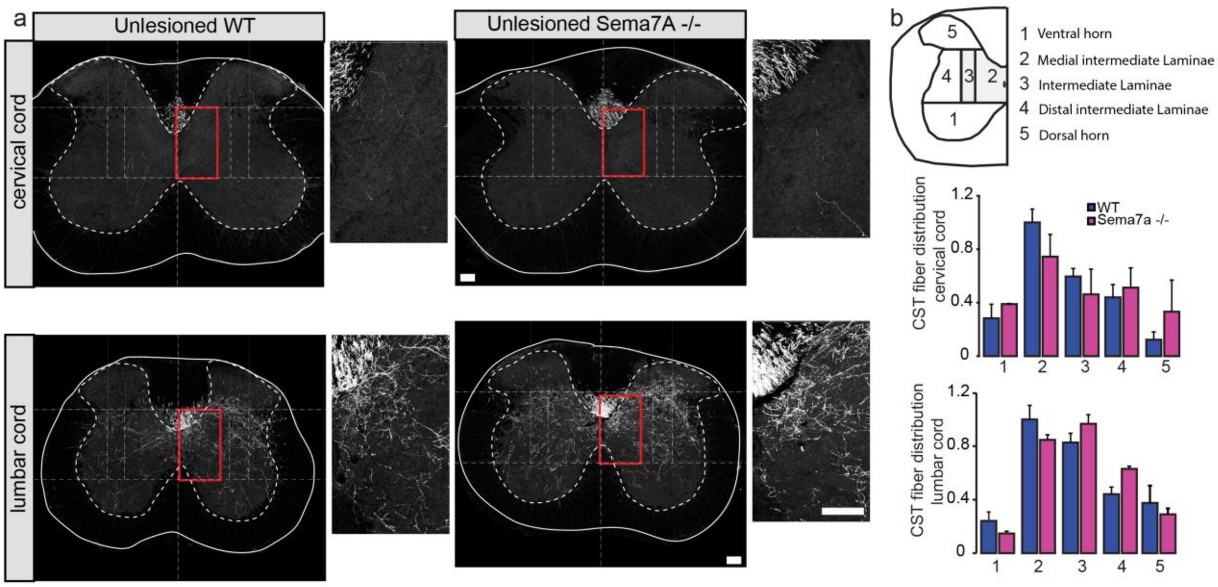

ESM_2 The density and distribution of hindlimb corticospinal collaterals is maintained in the cervical and lumbar spinal cord of Sema7A deficient mice

a Confocal images and regional analysis of collateral projections in the cervical (top) and lumbar (bottom) cord of WT (left) and Sema7A-/- (right) mice. Areas boxed in red are magnified 2.5 times in the insets.

b Schematic representation of the regional quantifications of the corticospinal projection in the different areas of the spinal cord (top) and quantification of the relative CST fiber distribution in the cervical and lumbar cord (middle and bottom panels; cervical cord p-values: 1=0,7000; 2=0,4000; 3=>0,9999; 4=>0,9999; 5=0,7000; lumbar cord p-values: 1=0,7000; 2=0,4000; 3=0,4000; 4=0,1000; 5=>0,9999). n=5 per group; Mann-Whitney-U-Test for every region and scale bars equal 100μm.

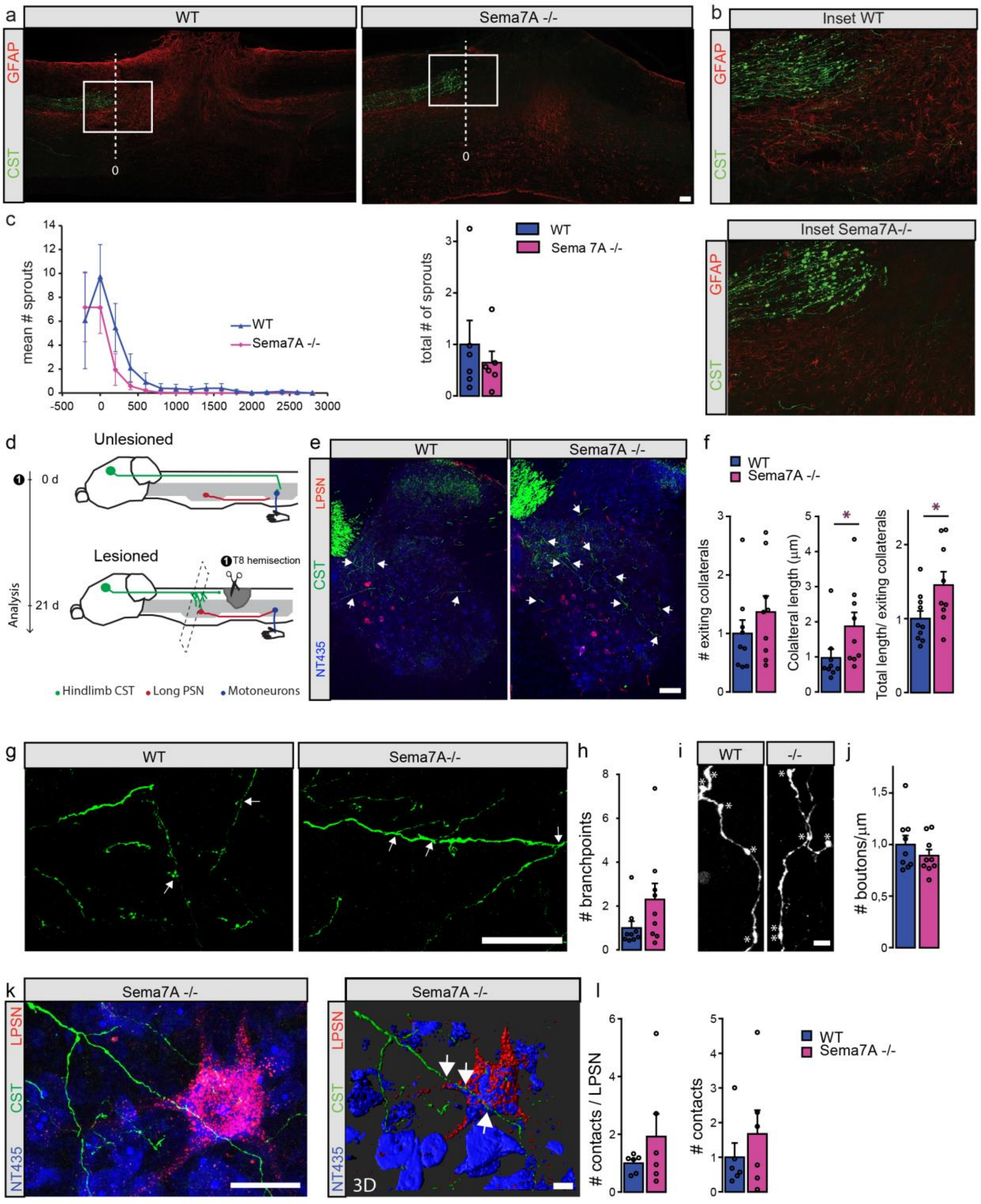

ESM_3 Genetic disruption of Sema7A signaling does not change axonal regeneration or overall remodeling of the corticospinal tract following spinal cord injury

a Confocal images of longitudinal sections of the spinal cord showing the corticospinal tract 3wks after spinal cord lesion (CST: green, GFAP: red). Dotted line indicates the level of retraction bulbs and the 0 line from which regeneration was evaluated.

b Higher magnification insets (3.5X) of the boxed areas in (**a**).

c Quantification of the normalized number of sprouts at different distances from the 0 line defined from the level of retraction bulbs (top; Two-way ANOVA p-value=0,9687) and cumulative normalized number of sprouts at 3wks after lesion (bottom, n=6 mice per group; p-value=0,5146).

d Timeline of the experiment and scheme of the rewiring of the hindlimb corticospinal tract following spinal cord injury.

e Coronal confocal images of hindlimb CST collaterals entering the cervical spinal cord (arrowheads) 3 weeks following T8 dorsal bilateral hemisection in WT (left panel) and Sema7A deficient (right panel) mice.

f Quantification of the number of exiting hindlimb CST collaterals (left panel, p-value=0,1614), collateral length (middle panel, p-value=0,0382) and length/exiting collaterals (right panel, p-value=0,0236) at 3 weeks following T8 dorsal bilateral hemisection in Sema7A deficient and WT mice (n=10 animals per group).

g Confocal images of representative branches (arrows) of the hindlimb CST collaterals in WT (left panel) and Sema7A deficient (right panel) mice.

h Quantification of the number of branch points on hindlimb CST collaterals (n=10 animals per group, p-value=0,0642).

i Representative confocal images showing putative synaptic boutons (asterisks) on hindlimb CST collaterals at 3 weeks following spinal cord injury in WT (left panel), Sema7A deficient (right panel).

j Quantification of the bouton density in WT and Sema7A deficient mice (n=10 animals per group, p-value=0,1629).

k Confocal image (left panel) of contacts between a long propriospinal neuron (red) and a CST collateral (green) and 3D Imaris reconstruction of the contact points (right panel). Arrows indicate points of contacts.

l Quantification of the number of contacts per long propriospinal neuron (p-value=0,2989) and total number of contacts (p-value=0,4229)(n = 6 animals per group). Mean ± SEM.

Scale bar in (**a** represents 200µm. Scale bar in (**e,g**) equals 100 µm. Scale bar in (**i**) equals 10 µm. Scale bars equals 25µm in **k** (left panel) and 15 µm in **k** (right panel). If not stated otherwise statistic is done with an unpaired two-sided t-Test for comparisons of WT versus Sema7A deficient mice.

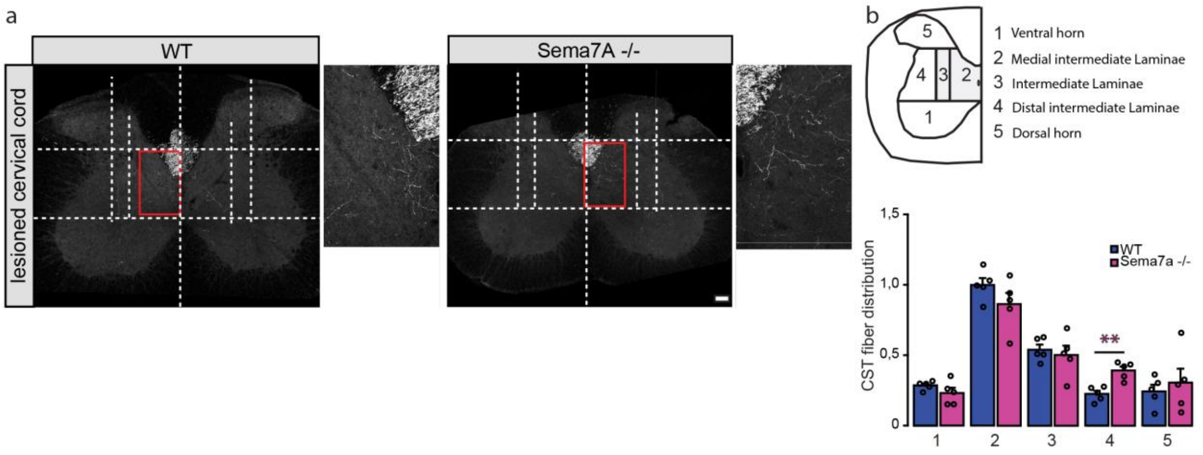

ESM_4 The distribution of hindlimb corticospinal collaterals is maintained in the lesioned cervical cord of Sema7A deficient mice

a Confocal images of the cervical spinal cord and regional analysis of collateral projections in the cervical cord of lesioned WT (left) and Sema7A deficient (right) mice. Areas boxed in red are magnified 2.5 times in the insets.

b Schematic representation of the regional quantification of the corticospinal projections in the different areas of the spinal cord (top) and quantification of the relative CST fiber distribution in the lesioned cervical cord (p-values: 1=0,3095, 2=0,2222, 3=>0,9999, 4=0,0079, 5=0,8413; Mann-Whitney-U-Test for comparison of every area). n=5 per group and scale bars equal 100μm.

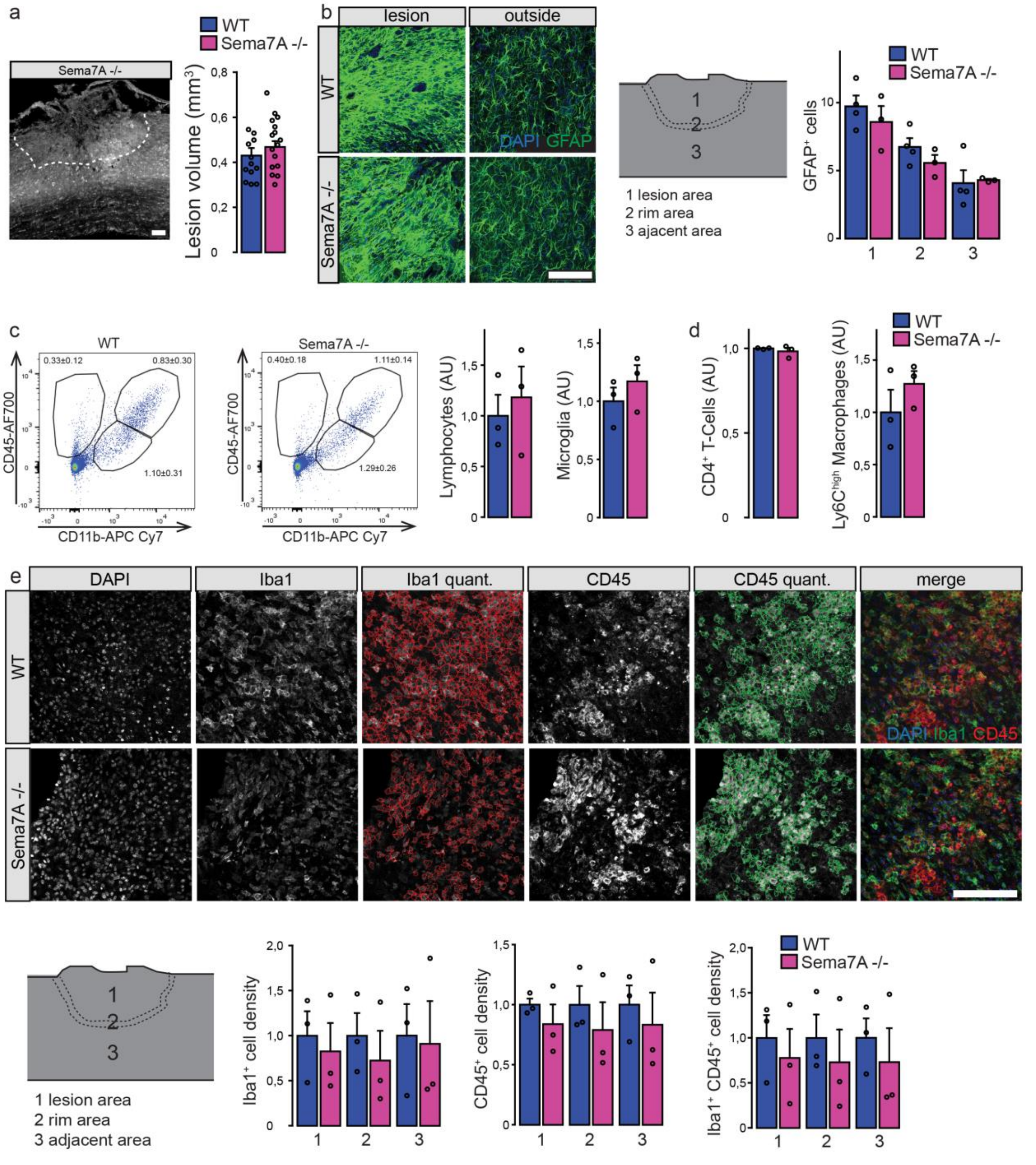

ESM_5 Genetic disruption of Sema7A signaling does not result in increased lesion volume or changes in inflammation

a Fluorescence image of a longitudinal section of a spinal cord lesion (dashed lines outline the lesion border) and quantification of lesion volume between Sema7A-deficient (pink bars; n=15) and Sema7A-competent mice (blue bars; n=13; p-value=0,3162).

b Characterization of the glial response following spinal cord injury in the lesion area and peri lesion (outlined in the graph in the middle panel; p-values: 1=0,6286, 2=0,4000, 3=0,4000). (n=3 per groups). Plotted are GFAP+ cells per 10000µm^2.

c Flow cytometric analysis of CD45 and CD11b positive immune cell population in WT and Sema7A deficient mice and quantification of the number of lymphocytes and microglia (n = 3 both groups, each normalized to WT, p-value$_{Lymphocytes}$=0,9999; p-value$_{Microglia}$=0,4000).

d Quantification of CD4$^+$ T cells and Ly6C^{high} macrophages following FACS analysis in WT and Sema7A deficient mice (n=3 both groups, each normalized to WT, p-value$_{CD4}$=0,6000, p-value$_{Macrophages}$=0,4000).

e Confocal pictures and quantifications of the immune cell infiltration in WT and Sema7A deficient mice using DAPI, Iba1 and CD45 marker in Sema7A deficient (right; n=3) and Sema7A-competent (left; n=3; normalized to WT) in the lesion area, perilesion and outside the lesion as depicted in the spinal cord scheme (p-values: Iba1$^+$: 1=0,9999, 2=0,4000, 3=0,9999; CD45$^+$: 1=0,7000, 2=0,4000, 3=0,7000; Iba1$^+$ CD45$^+$: 1=0,9999, 2=0,4000, 3=0,7000). All scale bars represent 100µm. All statistics is done with a Mann-Whitney-U-Test for comparison of WT and Sema7a deficient animals.

		PC1	PC2
**Run Parameters**	Run_Duration_(s)_Mean	-0.1376928	0.09236275
	Run_Average_Speed_(cm/s)_Mean	0.1316989	-0.1023879
	Run_Maximum_Variation_(%)_Mean	-0.07160413	0.05441662
	OtherStatistics_Duration_Mean	-0.1453214	0.08622734
	OtherStatistics_Average_Speed_Mean	0.1305358	-0.1049986
	OtherStatistics_Maximum_Variation_(%)_Mean	-0.1195878	-0.05164744
	OtherStatistics_NumberOfSteps	-0.1369317	0.07004829
	OtherStatistics_Cadence	0.1222213	-0.09379755
	PrintPositions_RightPaws_Mean_(cm)	-0.1370818	-0.00594602
	PrintPositions_LeftPaws_Mean_(cm)	-0.104979	0.05028466
**Forelimb Parameters**	FP_Stand_(s)_Mean	-0.1230347	0.1096476
	FP_StandIndex_Mean	-0.07612229	0.1349018
	FP_MaxContactAt_(%)_Mean	-0.0376092	-0.03497159
	FP_MaxContactArea_(cm^{2})_Mean	-0.0340216	0.1389527
	FP_MaxContactMaxIntensity_Mean	-0.05505419	0.1173273
	FP_MaxContactMeanIntensity_Mean	-0.07115606	0.06121838
	FP_PrintLength_(cm)_Mean	0.00233667	0.08982271
	FP_PrintWidth_(cm)_Mean	0.00089433	0.06808972
	FP_PrintArea_(cm^{2})_Mean	-0.01960663	0.1378974
	FP_MaxIntensityAt_(%)_Mean	-0.03753477	-0.00093528
	FP_MaxIntensity_Mean	-0.0594522	0.1269936
	FP_MinIntensity_Mean	-0.03005432	-0.1273
	FP_MeanIntensity_Mean	-0.07873485	0.07446444
	FP_MeanIntensityOfThe15MostIntensePixels_Mean	-0.0573561	0.1331343
	FP_Swing_(s)_Mean	0.0041639	0.08071737
	FP_SwingSpeed_(cm/s)_Mean	0.1061583	-0.09782246
	FP_StrideLength_(cm)_Mean	0.1390678	-0.04754986
	FP_StepCycle_(s)_Mean	-0.09077024	0.1130055
	FP_DutyCycle_(%)_Mean	-0.1462781	0.08349959
	FP_SingleStance_(s)_Mean	-0.03195374	0.1077032
	FP_InitialDualStance_(s)_Mean	-0.1474842	0.07253015
	FP_TerminalDualStance_(s)_Mean	-0.1479764	0.06232328
	FP_BodySpeed_(cm/s)_Mean	0.1327702	-0.1004173
	FP_BodySpeedVariation_(%)_Mean	-0.09577617	0.04089405
**Hindlimb Parameters**	HP_Stand_(s)_Mean	-0.04902299	0.1733664
	HP_StandIndex_Mean	-0.1010472	0.09771637
	HP_MaxContactAt_(%)_Mean	0.02816352	-0.075542
	HP_MaxContactArea_(cm^{2})_Mean	0.02762474	0.1648533
	HP_MaxContactMaxIntensity_Mean	0.03804275	0.173383
	HP_MaxContactMeanIntensity_Mean	0.0142984	0.1393947
	HP_PrintLength_(cm)_Mean	0.02496118	0.1678334
	HP_PrintWidth_(cm)_Mean	0.05621379	0.1584686
	HP_PrintArea_(cm^{2})_Mean	0.01304445	0.1676173
	HP_MaxIntensityAt_(%)_Mean	0.01233608	0.0304631
	HP_MaxIntensity_Mean	0.03209895	0.1795974
	HP_MinIntensity_Mean	-0.07943436	-0.134871
	HP_MeanIntensity_Mean	0.02363179	0.144976
	HP_MeanIntensityOfThe15MostIntensePixels_Mean	0.03155205	0.1735324
	HP_Swing_(s)_Mean	-0.1096916	-0.07755345
	HP_SwingSpeed_(cm/s)_Mean	0.1348834	0.01652878
	HP_StrideLength_(cm)_Mean	0.1182174	-0.06418713
	HP_StepCycle_(s)_Mean	-0.1310362	0.07509746
	HP_DutyCycle_(%)_Mean	0.01594417	0.1735892
	HP_SingleStance_(s)_Mean	0.0089278	0.1348756
	HP_InitialDualStance_(s)_Mean	-0.07108196	0.1447132
	HP_TerminalDualStance_(s)_Mean	-0.07605059	0.1400615
	HP_BodySpeed_(cm/s)_Mean	0.1367207	-0.09301926
	HP_BodySpeedVariation_(%)_Mean	-0.08632861	0.01726901
**Step Sequence (SS) Parameters**	StepSequence_NumberOfPatterns	-0.00645039	0.1476359
	StepSequence_CA_(%)	0.06073391	0.03427481
	StepSequence_CB_(%)	0.04912191	0.03646949
	StepSequence_AA_(%)	0.06341821	0.04100541
	StepSequence_AB_(%)	-0.07401928	-0.03492282
	StepSequence_RA_(%)	-0.06537864	-0.03976231
	StepSequence_RB_(%)	-0.0943856	-0.1105229
	StepSequence_RegularityIndex_(%)	0.1298639	0.09253915
**Base of Support (BoS)**	BOS_FrontPaws_Mean_(cm)	-0.07110151	0.06811077
	BOS_HindPaws_Mean_(cm)	0.00092704	0.09970959
**Phase Dispersions**	PhaseDispersions_RF->LH_Mean	-0.06187374	0.00578943
	PhaseDispersions_RF->LH_CStat_Mean	0.04730213	0.03715065
	PhaseDispersions_RF->LH_CStat_R	0.05987698	-0.00141208
	PhaseDispersions_LF->RH_Mean	-0.08482359	-0.02937122
	PhaseDispersions_LF->RH_CStat_Mean	0.01030935	-0.01355798
	PhaseDispersions_LF->RH_CStat_R	0.1180044	0.07467159
	PhaseDispersions_LH->RH_Mean	0.1038088	0.04381169
	PhaseDispersions_LH->RH_CStat_Mean	0.02889138	0.00658021
	PhaseDispersions_LH->RH_CStat_R	0.1229716	0.07310999
	PhaseDispersions_LF->RF_Mean	0.03082277	-0.00445281
	PhaseDispersions_LF->RF_CStat_Mean	0.02723167	0.04021845
	PhaseDispersions_LF->RF_CStat_R	0.07742746	-0.05765828
	PhaseDispersions_RF->RH_Mean	0.09387346	0.02145243
	PhaseDispersions_RF->RH_CStat_Mean	-0.1125157	-0.09303384
	PhaseDispersions_RF->RH_CStat_R	0.140511	0.04976415
	PhaseDispersions_LF->LH_Mean	0.07125456	-0.0048527
	PhaseDispersions_LF->LH_CStat_Mean	-0.1352022	-0.07330392
	PhaseDispersions_LF->LH_CStat_R	0.08006381	-0.00799412

		PC1	PC2
**Couplings**	Couplings_RF->LH_Mean	0.08743664	0.01872788
	Couplings_RF->LH_CStat_Mean	0.05318331	0.03615011
	Couplings_RF->LH_CStat_R	0.06583817	0.01823499
	Couplings_LF->RH_Mean	0.09414825	0.07033666
	Couplings_LF->RH_CStat_Mean	0.01734719	-0.0128642
	Couplings_LF->RH_CStat_R	0.1345004	0.07564358
	Couplings_LH->RF_Mean	0.1371573	0.03726139
	Couplings_LH->RF_CStat_Mean	-0.059385	-0.0282503
	Couplings_LH->RF_CStat_R	0.1285335	0.03806266
	Couplings_RH->LF_Mean	0.1179379	0.05890562
	Couplings_RH->LF_CStat_Mean	-0.0207939	-0.0004613
	Couplings_RH->LF_CStat_R	0.1159825	0.07909224
	Couplings_LH->RH_Mean	0.02279844	-0.0167655
	Couplings_LH->RH_CStat_Mean	0.04793951	0.02599924
	Couplings_LH->RH_CStat_R	0.1250624	0.08011004
	Couplings_LF->RF_Mean	0.02415569	0.0402187
	Couplings_LF->RF_CStat_Mean	0.02106645	0.0405869
	Couplings_LF->RF_CStat_R	0.08286373	-0.0607499
	Couplings_RH->LH_Mean	0.01025283	0.01727813
	Couplings_RH->LH_CStat_Mean	-0.0157522	0.0412498
	Couplings_RH->LH_CStat_R	0.09668155	0.07967905
	Couplings_RF->LF_Mean	-0.0291969	-0.0260607
	Couplings_RF->LF_CStat_Mean	-0.0344673	-0.022803
	Couplings_RF->LF_CStat_R	0.07647578	-0.0850557
	Couplings_RF->RH_Mean	-0.0751287	-0.0726491
	Couplings_RF->RH_CStat_Mean	-0.1165434	-0.0921562
	Couplings_RF->RH_CStat_R	0.1421636	0.04834453
	Couplings_LF->LH_Mean	-0.1243758	-0.0846585
	Couplings_LF->LH_CStat_Mean	-0.1360162	-0.0716617
	Couplings_LF->LH_CStat_R	0.086144	-0.0062264
	Couplings_RH->RF_Mean	0.01314412	0.05683777
	Couplings_RH->RF_CStat_Mean	0.1075359	0.09091037
	Couplings_RH->RF_CStat_R	0.1342578	0.08613191
	Couplings_LH->LF_Mean	0.01387836	-0.0004367
	Couplings_LH->LF_CStat_Mean	0.1295235	0.07503747
	Couplings_LH->LF_CStat_R	0.1284985	0.02819099
**Support**	Support_Zero_(%)	0.04418781	-0.1161072
	Support_Single_(%)	-0.0417775	-0.1679333
	Support_Diagonal_(%)	0.1471613	0.03924239
	Support_Girdle_(%)	-0.1096056	-0.0685475
	Support_Lateral_(%)	-0.0624786	-0.0574899
	Support_Three_(%)	-0.0813276	0.126396
	Support_Four_(%)	-0.0663396	0.1221306

ESM_6 Detailed PCA factor loadings (from Figure 5) for the Catwalk analysis in WT and Sema7A-/- following spinal cord injury.

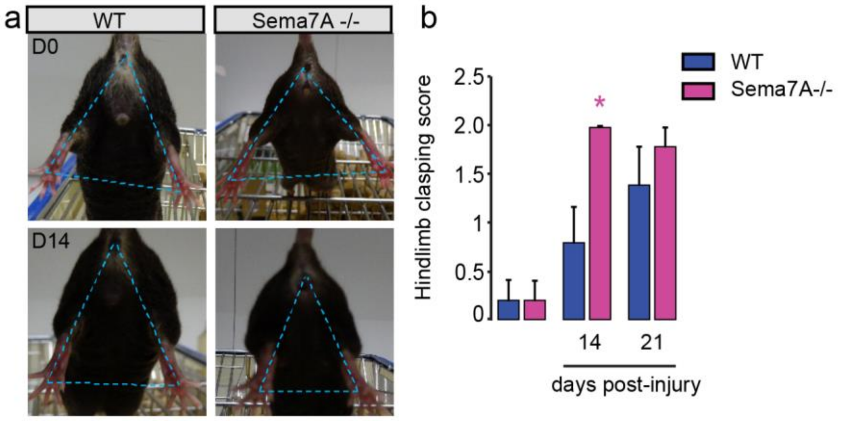

ESM_7 Hindlimb clasping analysis following spinal cord injury in WT and Sema7A-/- mice.

a Images of the hindlimb clasping test performed in WT and Sema7A-/- mice. The dashed light blue triangles highlight differences in the hindlimb spread angle.

b Average hindlimb clasping score was evaluated in WT and Sema7A-/- mice following spinal cord injury.
